# Carpal Tunnel Release Surgery and Venous Hypertension in Early Hemodialysis Patients without Amyloid Deposits

**DOI:** 10.1155/2013/481348

**Published:** 2013-11-05

**Authors:** Ismail Kocyigit, Aydin Unal, Ahmet Guney, Ertugrul Mavili, Kemal Deniz, Merva Kocyigit, Murat Sipahioglu, Eray Eroglu, Bulent Tokgoz, Ali Ihsan Gunal, Oktay Oymak

**Affiliations:** ^1^Department of Nephrology, Erciyes University Medical Faculty, 38039 Kayseri, Turkey; ^2^Department of Orthopedics and Traumatology, Erciyes University Medical Faculty, 38039 Kayseri, Turkey; ^3^Department of Radiology, Erciyes University Medical Faculty, 38039 Kayseri, Turkey; ^4^Department of Pathology, Erciyes University Medical Faculty, 38039 Kayseri, Turkey; ^5^Department of Neurology, Erciyes University Medical Faculty, 38039 Kayseri, Turkey; ^6^Department of Internal Medicine, Erciyes University Medical Faculty, 38039 Kayseri, Turkey; ^7^Department of Nephrology, Kayseri Training and Research Hospital, 38010 Kayseri, Turkey

## Abstract

*Aim*. Carpal tunnel syndrome (CTS) is one of the frequent problems of the patients who underwent hemodialysis (HD). The role of venous hypertension due to arteriovenous fistula (AVF) has not been clarified completely; therefore, we aimed to investigate the role of venous hypertension due to AVF in hemodialysis patients who had CTS. *Patients and Methods*. We included 12 patients who had been receiving HD treatment for less than 8 years and the newly diagnosed CTS patients with the same arm of AVF. All patients were diagnosed clinically and the results were confirmed by both nerve conduction studies and electromyography. Open carpal tunnel release surgery was performed on all of them. Venous pressure was measured in all patients before and after two weeks of surgery. *Results*. There were significant differences before and after the surgery with regard to pressures (*P* > 0.05). After the surgery, all carpal ligament specimens of the patients were not stained with Congo red for the presence of amyloid deposition. *Conclusion*. Increased venous pressure on the same arm with AVF could be responsible for CTS in hemodialysis patients. Carpal tunnel release surgery is the main treatment of this disease by reducing the compression on the nerve.

## 1. Introduction

Peripheral nerve entrapment is one of the common problems in patients with end stage renal disease, especially in those receiving hemodialysis. The most encountered form is median nerve entrapment entitled carpal tunnel syndrome that is seen between the rates of 9% and 32% in HD population [1–6]. The syndrome has been increasingly reported in HD patients over the years, since its first description in 1975 [1]. Many authors focused on the underlying etiology and pathogenesis of this syndrome. Although there is no precise cause defined this syndrome, it has been demonstrated that many factors are attributed to developing this syndrome in HD patients. These factors are often related to vascular access [2], fluid overload [3], duration of hemodialysis [6, 7], accumulation of *β*-2 microglobulin amyloid fibrils [8, 9], and arterial calcifications [10]. Recently, investigators have emphasized the role of amyloid accumulation based on the cases with long hemodialysis duration and presence of *β*-2 microglobulin associated amyloid fibrils in biopsy specimens after surgery for CTS [8, 9].

However, early developed and amyloid negative CTS cases had been declared by many authors, and the role of amyloid accumulation became suspicious to elucidate the etiology. Conformably, predilection of CTS on the same arm with fistula was taken into consideration in the role of vascular access. Based upon these findings, another important hypothesis was unveiled that consists of the compression of arteriovenous fistula, vascular steal syndrome edema due to venous outlet obstruction and also venous hypertension. Especially, increased symptoms during dialysis sessions promoted this hypothesis to be more accountable [1–4]. 

Although the role of venous hypertension has been demonstrated in earlier reports, the underlying mechanism remained unclear. Therefore, we aimed to clarify the role of the venous hypertension for the etiology of the hemodialysis patients with CTS. In this study, we reported our experience in 12 HD patients with unilateral CTS on the fistula arm and its close relation to venous hypertension.

## 2. Patients and Method

### 2.1. Study Population

The study included patients referred to our university hospital with signs and symptoms of the CTS confirmed by electrodiagnostic tests including nerve conduction studies and electromyography. We excluded patients aged less than 18 years or patients who had already been treated for CTS. In addition patients, who underwent hemodialysis for more than 8 years were excluded because CTS has the average time to onset being approximately 8–10 years after the initiation after dialysis [8]. The study was approved by the local ethics committee. Patients gave written informed consent.

### 2.2. Measurement of Venous Pressure

First, a Doppler ultrasound was performed and the outflow vein was identified. Then, a tourniquet was applied for passive vein congestion. Thereafter; the outflow vein was punctured with a 16-gauge sheath needle (Abbott) in a retrograde fashion against the flow direction under local anesthesia. Then, a 5-F introducer sheath (Terumo, Tokyo, Japan) was placed into the vein. And images were obtained to identify the fistula. After that a 5-F multipurpose catheter (William Cook Euro pe ApS, Bjaeverskov, Denmark) and a hydrophilic-coated and steerable 0.035-inch guidewire (Terumo) were passed across the fistula and placed into the artery. The guidewire was withdrawn and a transducer (Pressure Monitoring Set, Bicakcilar, Istanbul, Turkey) was attached to the catheter. All pressures were measured using a multipurpose monitor (KMA 900, PETAs, Turkey). The pressures were measured at the venous, fistula, and arterial parts. Calculations were repeated three times at each level and the mean values were recorded. At each level the systolic, diastolic, and mean pressures were measured. This procedure was repeated one week after operation.

The reduction in pressure (i.e., venous pressure (ΔVP)) was calculated following the next formula

(1) ΔP=[(pressure before the operation) −(pressure after the operation)] ×((pressure before the operation)×100)−1.

### 2.3. Open Carpal Tunnel Release Surgery

Using the described techniques below the full release of the median nerve has been performed.

Open carpal tunnel release is technically straightforward and this technique does not required additional instruments. The procedure is performed with the patient supine and the extremity on a hand table. A general anesthesia is performed. The extremity is exsanguinated and the tourniquet is not used because of the AV fistula. We can use S-shaped incision which extended from the distal palm to the proximal forearm allowing full exposure of the median nerve at the wrist and forearm. In an effort to decrease the risk for injury to the palmar cutaneous branches of the median and ulnar nerves, the incision for the open carpal tunnel release is made along the ring finger axis. Although there is not a true internervous zone between the palmar cutaneous branch of the median nerve and the palmar cutaneous branch of the ulnar nerve, an incision in the ring finger axis should result in the injury of fewer nerve fibers [11, 12].

We can expect good relief of neurologic symptoms, although grip-strength weakness and pillar pain have led to a search for alternative procedures.

### 2.4. Histopathological Examination

Materials were available for histopathologic examination for each of them. All specimens were fixed in 4% buffered formaldehyde, decalcified in 5% formic acid for one to two weeks, embedded in paraffin, and cut into serial slides. The slides were stained by hematoxylin eosin and alkaline Congo red [13]. When detected, amyloid was typed by an avidin-biotin-peroxidase complex and anti-b2m (dilution 1/100; Dako, Copenhagen, Denmark), anti-P component (dilution 1/500; Dako), antiamyloid protein A (dilution 1/500; Dako), antiprealbumin (dilution 1/200; Dako), anti-kappa (dilution 1/10,000; Bio-Yeda Ltd, Rehovot, Israel), and antilambda (dilution 1/10,000; Bio-yeda, Rehovot, Israel) antibodies.

### 2.5. Electrodiagnostic Tests

Nerve conduction studies were performed using a Counterpoint MK2 (Dantec, Copenhagen, Denmark). The median, ulnar, peroneal, and tibial motor nerves and median, ulnar, and sural sensory nerves were evaluated using standard conduction techniques. The distance between the recording electrode and stimulation was 8 cm in all of the compound muscle action potential (CMAP) recordings and was 14 cm for the sensory nerve action potential (SNAP) recordings. The F waves of all the motor nerves and the H reflex were also evaluated. Skin temperature was maintained at 32°C or above in the upper extremity nerves and 30°C or above in the lower extremity nerves. PN was diagnosed and graded from I to IV based on the criteria of our laboratory, a modification of the diabetes control and complication trial (DCCT) research group [14]. CTS was diagnosed when the findings met at least one of the following items regardless of the presence or absence of a PN: (1) ratio of median sensory latency of a 7 cm wrist segment to a 7 cm palm segment > 2.0, (2) ratio of distal latency of a median SNAP to that of an ulnar SNAP > 1.2, (3) ratio of the amplitude of a median sensory SNAP with wrist stimulation to that with palm stimulation < 0.5, (4) ratio of the distal latency of a median CMAP to that of an ulnar CMAP > 1.5, and (5) ratio of the amplitude of a median CMAP to that of an ulnar CMAP < 0.6. These cut off values are obtained from the 50 healthy volunteers aging from 26 to 75 (mean age, 55.2 ± 12.0 yr).

### 2.6. Statistical Analysis

SPSS 16.0 software was used for the statistical analysis. Kolmogorov-Smirnov test was used for normality analysis of quantitative variables. Continuous variables with normal distribution were presented as mean ± standard deviation. Statistical analysis for the parametric variables was performed by the paired *t*-test. Median value was used where normal distribution is absent. The Wilcoxon signed-rank test was used to compare nonparametric variables. The correlation analysis was evaluated by the Pearson's correlation test. A *P* value <0.05 was considered statistically significant.

## 3. Results

Characteristics of patients are seen in Table 1. 

Consider

(2) ΔP=|[(pressure before the operation) −(pressure after the operation) ×(pressure before the operation)−1]×100|.

None of the biopsy specimens, which were obtained from 12 patients, was stained with Congo red for the presence of amyloid deposition. 

**Figure 1:**
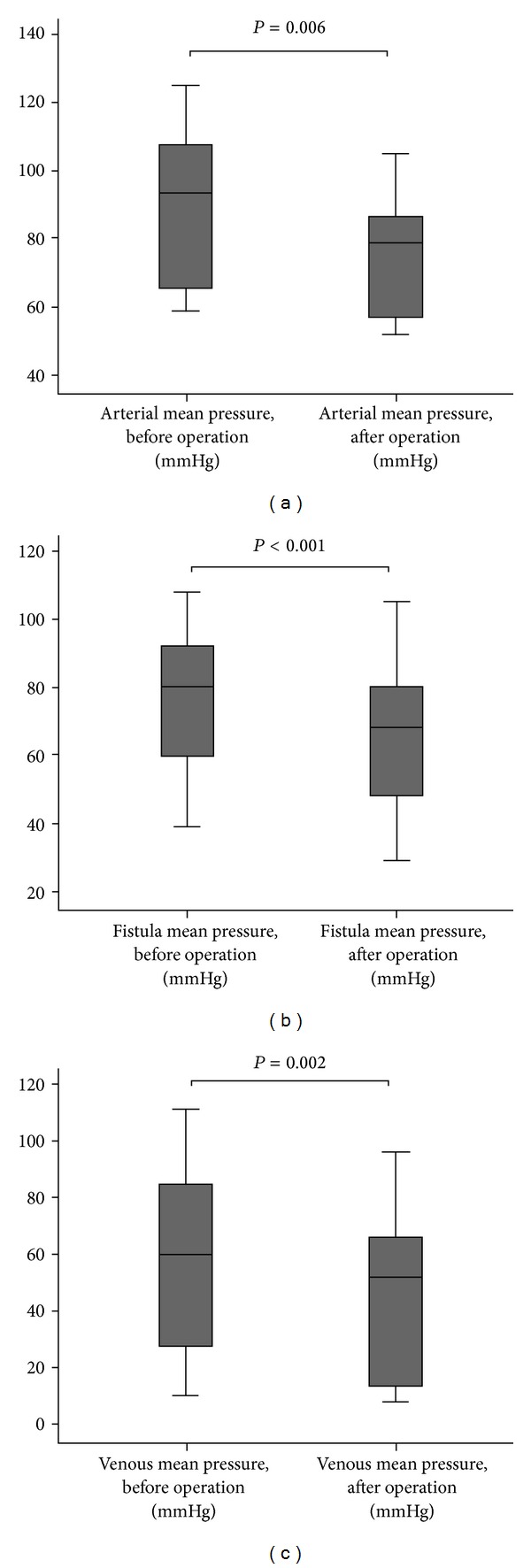
Comparison of arterial mean pressure, venous mean pressure, and fistula mean pressure before and after release surgery.

Table 2 shows comparison of the pressures in different localizations before and after the operation. There were significant differences between the 2 time periods with regard to pressures (*P* < 0.05). Pressures in all localizations were significantly lower after the operation than those before the operation (Figure 1).

Δ*P* in all localizations was not correlated with levels of serum *β*-2 microglobulin level, high sensitive C-reactive protein, intact parathormone, dialysis duration, BMI, and systemic systolic and diastolic blood pressures (*P* > 0.05). Similarly, serum *β*-2 microglobulin levels were not correlated with dialysis duration (*P* > 0.05).

## 4. Discussion

In terms of CTS, risk was shown to be increased in HD patients compared to the general population [15]. In addition to the burden of chronic kidney disease and hemodialysis, impaired health-related quality of life and socioeconomic deprivation due to CTS have been well established [16, 17]. Moreover, surgical treatment is widely accepted in HD patients with CTS; thus, authors have focused on the underlying mechanism to prevent CTS in HD patients before its occurrence [1, 4, 18–22].

CTS was first described in the HD population in 1975 by Warren and Otieno. It has been reported that increased venous pressure due to vascular access could be responsible for CTS in HD patients [1]. Since then, many factors have been proposed for the development of CTS, particularly *β*2 microglobulin amyloid accumulation in long term dialysis patients independently from vascular shunt. However, the early development of CTS in HD patients could not be explained by amyloid accumulation [8, 9]. Therefore, we aimed to investigate venous hypertension and its close relation with CTS in HD patients. Based on previous studies, we hypothesized that increased venous hypertension contributes to the extravasation of fluid and leads to extrinsic compression of the median nerve that results in CTS. We reviewed 12 patients with CTS in the same arm as the fistula who were treated with open surgery. Additionally, pathologic examination did not reveal *β*-2 microglobulin amyloid accumulation in the surgical specimens. After surgery, decreased venous pressure on the affected limb was demonstrated for the first time in this study setting.

The carpal tunnel is a narrow area circumscribed by the carpal ligament superiorly and by the carpal bones inferiorly. The median nerve that is located in this tunnel may be affected by increased pressure throughout the passage or compressed by surrounding structures. After the observation of CTS in HD patients in the same arm as the AVF, researchers addressed the role of vascular shunt and its complications [19]. There are two main hypotheses on the development of CTS : (I) venous hypertension and congestion of the distal limb result in compression on the median nerve inside the carpal tunnel [1, 4] and (II) a vascular steal phenomenon resulted in ischemic neural injury and nerve dysfunction [4, 20]. The occurrence of CTS symptoms during hemodialysis sessions and early development of CTS after fistula creation support these vascular hypotheses [1, 4, 16, 18]. We hypothesized that increased venous hypertension due to fistula results in congestion and leakage of fluid throughout the carpal tunnel and this could be responsible for increased pressure on the median nerve. Therefore, chronic irritation of the ligament might induce thickening of the carpal ligament as a consequence. In a vicious cycle, narrowing of the carpal canal might contribute to an increased in venous tension. Despite our results, our hypothesis may be insufficient to explain the previously reported CTS cases associated with *β*-2 microglobulin composed amyloid fibrils [8, 9]. Otherwise, the role of time-dependent *β*-2 microglobulin accumulation and its relation to CTS patients with long term dialysis duration are still under debate, due to the gradual occurrence of vascular complications that was exposed by researchers [1, 4, 19–21].

One early study on CTS in HD patients [1] suggested that side-to-side type anastomosis could be responsible for venous hypertension and hand edema; conversely, we did not observe hand edema on the fistula hand affected with CTS and all of our patients had end-to-side type anastomosis. Recent studies showed that the type of anastomosis was not associated with the development of CTS [23, 24].

In contrast to studies on vascular phenomenon, Charra et al. reported that the limb affected by CTS was not related to the location of the arteriovenous fistula and amyloid positive CTS cases were also noticed predominantly to have shoulder pain; these relations were confirmed in many studies [8, 23, 24]. Interestingly, no complaints of shoulder pain were made by our patients and the pathologic examination of surgery specimens was negative for amyloid fibrils. Therefore, shoulder pain might be a clue in amyloid related arthropathy [24, 25]. The amyloid theory on CTS has been supported by consecutive reports of contralateral and bilateral cases independent of fistula creation [9, 20, 26]. On the other hand, McClure et al. stated that CTS is one of the clinical entities in patients receiving long term hemodialysis that is associated with amyloid deposition in the perineural and periarticular structures [27]. Moreover, the prevalence of CTS correlates with the duration of hemodialysis since it is thought that the accumulation of *β*-2 microglobulin amyloid fibrils is time dependent [8, 28]. *β*-2 microglobulin levels were reported to be increased in chronic renal failure, significantly in HD patients compared to peritoneal dialysis patients [29]. Nomoto et al. reported only 7 CTS cases in 5050 peritoneal dialysis patients and only 2 biopsies of the patients were positive for amyloid accumulation so they concluded that PD minimizes the emergence of CTS [30]. However, in a recent study, it was demonstrated that there was no correlation with *β*-2 microglobulin levels and CTS [22]. In contrast, Chanard et al. demonstrated that CTS cases were lowered in HD patients using *β*-2 microglobulin permeable membranes compared to less permeable ones [31]. According to these studies, *β*-2 microglobulin amyloid fibril deposition alone could not explain the pathogenesis of CTS in HD patients, but it may be a contributing factor, especially in late cases of CTS. Possibly, CTS might be triggered exclusively by *β*-2 microglobulin amyloidosis due to increased venous pressure near the AVF in late cases. Obviously, further studies with a larger number of patients and a longer observation period are needed to clarify the temporal realtionship with increased venous pressure and accumulation of amyloid fibrils near the fistula.

In addition to CTS symptoms, the diagnosis of CTS in HD patients requires electrodiagnostic tests to exclude both uremic and diabetic neuropathies. Since diabetic nephropathy is the most common cause of end stage renal disease, diabetic neuropathy is a frequently encountered problem in this population. However, uremic and diabetic neuropathies are generally distal and symmetric; mononeuropathic types should be kept in mind as they could cause similar symptoms to CTS. In this regard, we used such electro diagnostic tests to exclude other neuropathy types and to diagnose CTS clearly in our HD patients [22, 32, 33].

There are possible limitations of this study. One of the limitations is the small number of patients included in the study. Larger studies should be conducted to clarify our findings. Another possible limitation could be the lack of bilateral CTS cases.

In conclusion, we demonstrated the role of venous hypertension in early CTS in HD patients in the absence of *β*-2 microglobulin amyloid fibrils. Increased venous pressure on the fistula hand might be a clue for CTS development in HD patients. Decreased venous pressure after open carpal tunnel release surgery was associated with the relief of CTS symptoms. As a result, increased venous hypertension due to vascular shunt might be an important reason for early CTS in HD patients.

## Figures and Tables

**Table 1:** Characteristics of 12 patients.

Age, years	63 ± 7
Gender, female/male	7/5
Dialysis duration, years	3.5 ± 1.3
Serum beta 2-microglobulin, mg/dL	21.0 ± 10.0
High sensitive C-reactive protein, mg/dL	7.8 (3.4–21.0)
Intact parathormone, pg/mL	247 (189–2484)
Hemoglobin, g/dL	12.5 ± 1.7
Systemic systolic blood pressure, mmHg	118 ± 16
Systemic diastolic blood pressure, mmHg	76 ± 12
Location of arteriovenous fistula	
Radial	10 (83.3%)
Brachial	2 (16.7%)
Cause of end stage renal disease	
Diabetes mellitus	4 (33.3%)
Hypertension	4 (33.3%)
Polycystic kidney disease	1 (8.3%)
Obstructive uropathy	1 (8.3%)
Unknown	2 (16.7%)

**Table 2:** Comparison of pressures in different localizations before and after operation.

Parameters	Before operation	After operation	Δ*P* (%)	*P* value
Arterial systolic pressure, mmHg	127 ± 48	106 ± 26	13.5	0.027
Arterial diastolic pressure, mmHg	64 ± 19	53 ± 20	17.1	0.027
Arterial mean pressure, mmHg	90 ± 22	74 ± 17	15.6	0.006
Fistula systolic pressure, mmHg	103 ± 35	92 ± 32	10.6	0.009
Fistula diastolic pressure, mmHg	58 ± 20	48 ± 22	17.6	0.027
Fistula mean pressure, mmHg	77 ± 23	66 ± 23	14.7	<0.001
Venous systolic pressure, mmHg	76 ± 41	65 ± 42	17.5	0.011
Venous diastolic pressure, mmHg	42 ± 28	33 ± 27	23.1	0.004
Venous mean pressure, mmHg	57 ± 35	46 ± 33	21.9	0.002

Δ*P* = |[(pressure before the operation)−(pressure after the operation)/pressure before the operation]  × 100|.
